# SILVOLIVE, a Germplasm Collection of Wild Subspecies With High Genetic Variability as a Source of Rootstocks and Resistance Genes for Olive Breeding

**DOI:** 10.3389/fpls.2020.00629

**Published:** 2020-05-28

**Authors:** Pablo Díaz-Rueda, Juan D. Franco-Navarro, Rita Messora, Joaquín Espartero, Carlos M. Rivero-Núñez, Pablo Aleza, Nieves Capote, Manuel Cantos, Jose L. García-Fernández, Alfonso de Cires, Angjelina Belaj, Lorenzo León, Guillaume Besnard, Jose M. Colmenero-Flores

**Affiliations:** ^1^Instituto de Recursos Naturales y Agrobiología, Spanish National Research Council (CSIC), Seville, Spain; ^2^Plant Physiology Laboratory, Dipartimento Sci Vita, Univ Modena & Reggio Emilia, Modena, Italy; ^3^Centro de Citricultura y Producción Vegetal, Instituto Valenciano de Investigaciones Agrarias, Moncada, Spain; ^4^Andalusian Institute of Agricultural and Fisheries Research and Training (IFAPA) Centro Las Torres, Seville, Spain; ^5^Departamento de Biología Vegetal y Ecología, Fac Biología, Univ de Sevilla, Seville, Spain; ^6^Andalusian Institute of Agricultural and Fisheries Research and Training (IFAPA) Centro Alameda del Obispo, Córdoba, Spain; ^7^CNRS-UPS-IRD, EDB, UMR 5174, Université Paul Sabatier, Toulouse, France

**Keywords:** *Olea europaea*, wild germplasm, molecular markers, genetic variability, vigor, branching, rootstock, grafting

## Abstract

Wild subspecies of *Olea europaea* constitute a source of genetic variability with huge potential for olive breeding to face global changes in Mediterranean-climate regions. We intend to identify wild olive genotypes with optimal adaptability to different environmental conditions to serve as a source of rootstocks and resistance genes for olive breeding. The SILVOLIVE collection includes 146 wild genotypes representative of the six *O. europaea* subspecies and early-generations hybrids. These genotypes came either from olive germplasm collections or from direct prospection in Spain, continental Africa and the Macaronesian archipelago. The collection was genotyped with plastid and nuclear markers, confirming the origin of the genotypes and their high genetic variability. Morphological and architectural parameters were quantified in 103 genotypes allowing the identification of three major groups of correlative traits including vigor, branching habits and the belowground-to-aboveground ratio. The occurrence of strong phenotypic variability in these traits within the germplasm collection has been shown. Furthermore, wild olive relatives are of great significance to be used as rootstocks for olive cultivation. Thus, as a proof of concept, different wild genotypes used as rootstocks were shown to regulate vigor parameters of the grafted cultivar “Picual” scion, which could improve the productivity of high-density hedgerow orchards.

## Introduction

The wild relatives of domesticated crops possess genetic diversity useful for developing more productive, nutritious and resilient crop varieties (Castaneda-Alvarez et al., 2016), and for preserving global food security against the serious threat of climate change (Vincent et al., 2013). Wild relatives of the domesticated olive tree (*Olea europaea* L.) are evergreen, drought tolerant, usually multi-stemmed small trees or large shrubs with very good adaptability to different environmental conditions (Médail et al., 2001; Green, 2002; Kassa et al., 2019). Wild olives grow in arid and semiarid regions at different altitudes and soil types, including those exposed to severe water deficit, salinity and low temperatures (Cantos et al., 2002; Baldoni et al., 2006; Klepo et al., 2013; Belaj et al., 2016; Chiappetta et al., 2017). This adaptability to adverse environmental conditions makes wild olive trees suitable to grow in marginal soils (e.g., at risk of desertification), to colonize deforested habitats or to rehabilitate devastated regions (Bekele, 2005; Kassa et al., 2019). Six olive subspecies have been recognized that occur in different natural distribution ranges in Europe, Africa, and Asia (Green, 2002): (1) *O. europaea* subsp. *europaea*, which includes wild types or oleasters [var. *sylvestris* (Mill.) Lehr] and the domesticated olive (var. *europaea*) that are common in the whole Mediterranean basin; (2) *O. e.* subsp. *cuspidata* (Wall. ex G. Don) Cif. distributed from South Africa to south-eastern Egypt and from the Middle East to India and China; (3) *O. e.* subsp. *laperrinei* (Batt. & Trab.) Cif. in the central Saharan mountains; (4) *O. e.* subsp. *maroccana* (Greut. & Burd.) P. Vargas et al. in south-western Morocco; (5) *O. e.* subsp. *cerasiformis* Kunk. & Sund. in Madeira; and (6) and *O. e.* subsp. *guanchica* P. Vargas et al. in the Canary Islands.

Besnard et al. (2007) showed through nuclear and plastid DNA data that the main wild progenitor of the cultivated olive (*O. e.* subsp. *europaea* var. *europaea*) is the wild Mediterranean olive, also known as oleaster (*O. e.* subsp. *europaea* var. *sylvestris*). Olive domestication from wild oleaster populations has involved the selection of a small number of desirable genotypes with bigger fruits, which were asexually propagated through cuttings. Such selection and propagation practices may contribute to reduce genetic diversity of the cultivated genepool (Rugini et al., 2011), but continuous hybridization events with local wild populations have, however, occurred during the long and ongoing domestication process (Besnard et al., 2013b, 2007). A higher genetic diversity is still observed in the wild genepool (Lumaret et al., 2004; Baldoni et al., 2009; Belaj et al., 2010; Besnard et al., 2013a; Chiappetta et al., 2017; Kassa et al., 2019). Wild olives therefore represent an important source of genes for crop improvement of resistance to abiotic stresses [e.g., salinity (Cantos et al., 2002), water deficit (Hernández-Santana et al., 2019), soil pollution (Murillo et al., 2005)], vigor (León et al., 2020), crop yield and quality (Hannachi et al., 2008; Baccouri et al., 2011; León et al., 2018), as well as for resistance to biotic factors such as the *Verticillium* wilt (Colella et al., 2008; Arias-Calderon et al., 2015b; Trapero et al., 2015; Jimenez-Fernandez et al., 2016). Wild olive genotypes have been tested in limited breeding studies, showing potential to shorten the juvenile period or to increase flower production (Klepo et al., 2014), to improve oil composition (Hannachi et al., 2008; León et al., 2018) and to improve resistance to soil-borne diseases (Arias-Calderon et al., 2015a).

An alternative and direct approach to take advantage of the gene-pool of wild germplasm is the use of selected wild genotypes as rootstocks, which greatly increases the efficiency of perennial crops. Rootstocks are commonly chosen for rooting capacity, abiotic and biotic stress resistance, and their ability to beneficially alter scion phenotypes such as precocity (early bearing), production, and fruit quality (Warschefsky et al., 2016). It is interesting to note that wild olive rootstocks were widely used in ancient cultivation systems (Barazani et al., 2014), while modern olive crops, unlike other perennial woody crops, use self-rooted cultivars. Reduction of vigor through the use of dwarf rootstocks is of particular interest in the cultivation of woody fruit trees. The main drawback of super-intensive olive orchards, also known as high-density hedgerow (HDH) system, is the difficulty to control the tree size to allow the movement of the harvesting machines (Tous et al., 2010). Cultivars used for HDH exhibit greater branching associated with smaller vigor parameters (Rosati et al., 2013). These features, which are difficult to gather in the same variety, determine that only a few traditional olive cultivars meet partially the low vigor requirement for HDH system, mostly “Arbequina,” “Arbosana,” and “Koroneiki” (Diez et al., 2016). Even these cultivars require tree size control by means of strict pruning and fertirrigation practices (Fernandez et al., 2013), which are expensive procedures. In addition, the HDH system excludes the possibility of using traditional cultivars of higher vigor, but of outstanding socioeconomic importance. Some studies indicate that certain olive cultivars used as rootstocks can regulate vigor traits like the canopy volume, stem section and production of the grafted scion (Baldoni and Fontanazza, 1990; Pannelli et al., 2002; Del Río and Caballero, 2006; Tous et al., 2012; Romero et al., 2014; Rugini et al., 2016). The use of wild genotypes to control the vigor of the grafted cultivar is also a matter of great interest (León et al., 2020), but no rootstocks of proven quality are currently available at either commercial or experimental levels.

It would therefore be desirable to have a catalog of wild genotypes representing most of the variability of the *O. europaea* species characterized for agronomical or eco-physiological traits of greatest interest. In the present study, we have characterized a germplasm collection of 146 olive genotypes representative of the six *Olea europaea* subspecies including hybrids. The collection has been genotyped and phenotyped for a number of morphological and developmental traits of interest. As a proof of concept, the ability of a number of wild genotypes to modify vigor features of the olive cultivar “Picual” has been addressed.

## Materials and Methods

### Plant Material and Culture Conditions

The wild olive germplasm collection, called SILVOLIVE, includes 146 genotypes obtained from seeds of mother trees prospected in their natural habitats or maintained in different Olive Germplasm Banks (WOGB-IFAPA Córdoba, WOGB-INRA Marrakech, and CEFE Montpellier; Table 1). The genotypes were *in-vitro* germinated from zygotic embryos of seeds from olive trees belonging to all subspecies of *Olea europaea* L. including hybrids (see Table 1 for detail): *O. e.* subsp. *guanchica* (GUA, ANA, HER, and BAR); *O. e.* subsp. *cerasiformis* (CER); *O. e.* subsp. *maroccana* (MAR); *O. e.* subsp. *cuspidata* (CUS, CEH); *O. e.* subsp. *europaea* (ACO, ACZ, AJA, AMK, AMS, AOU, APR, ARC, FRA, and TAM); and *O. e.* subsp. *laperrinei* (DHO) consisting of zygotic embryos of “Dhokar,” a Maghreb cultivated hybrid between *laperrinei* and *europaea* (Besnard et al., 2013a). The “Frantoio” (FRA) cultivar was the only elite olive variety exceptionally used as mother tree because of its potential interest in transmitting resistance to *Verticillium* wilt (Lopez-Escudero et al., 2004). The genotypes APR1 and ARC1 were previously obtained as seeds from salt-resistant wild olive trees present in Puerto Real (Cádiz, Spain) and Odiel (Huelva, Spain) salt marshes, respectively (Cantos et al., 2002).

**TABLE 1:** Origin and code of the 146 olive genotypes of the SILVOLIVE collection.

Mother tree						Number of genotypes
Subspecies	Lineage	Variety	Natural localization	Prospection	Acronym	
*europaea*	E1-*e*	“Frantoio”^Ω^	Tuscany, Italy	WOGB, Córdoba, Spain Acc. Number 80	FRA	4
		“Acebuche de Puerto Real” oleaster^Ω^	Puerto Real saline marshes, Cádiz, Spain	*In-vitro* germplasm collection M. Cantos (IRNAS, CSIC)	APR	1
		Unnamed oleaster^Ω^	Cádiz Mountains, Spain	WOGB, Córdoba, Spain Acc. Number W45	ACZ	10
		Unnamed oleaster^×^	Coria del Río, Seville, Spain	Coria del Río, Seville, Spain	ACO	5
		Unnamed oleaster^×^	Marrakech Mountains, Morocco	Marrakech, Morocco	AMK	11
		Unnamed oleaster^×^	Amskroud, Morocco	Amskroud, Morocco	AMS	9
	E2	“Raboconejo” oleaster^Ω^	Saltés Island in Odiel saline marshes, Huelva, Spain	*In-vitro* germplasm collection M. Cantos (IRNAS, CSIC)	ARC	1
		Unnamed oleaster^×^	Tamri, Morocco	Tamri, Morocco	TAM	5
		Unnamed oleaster^×^	Aourir, Morocco	Aourir, Morocco	AOU	12
	E3	Unnamed oleaster^Ω^	Sierra de Jaén, Spain	WOGB, Córdoba, Spain Acc. Number W69	AJA	6
*laperrinei* X *europaea*	E1-/1	“Dhokar”^×^	Tataouin zone, Tunisia	WOGB, Marrakech, Morocco Acc. Number Oct413	DHO	12
*guanchica*	M-*g1*	*guanchica*^Ω^	Tenerife, Canary Islands, Spain	WOGB, Córdoba, Spain Acc. Number W49	GUA	9
	M-*g1*	*guanchica*^×^	Tenerife, Canary Islands, Spain	Anaga, Tenerife, Spain	ANA	18
	M-*g1*	*guanchica*^×^	La Gomera, Canary Islands, Spain	Hermigua, La Gomera, Spain	HER	6
	M-*g2*	*guanchica*^×^	Gran Canaria, Canary Islands, Spain	Cañón del Cernícalo, Gran Canarias, Spain	BAR	9
*cerasiformis*	M-*c*	*cerasiformis*^Ω^	Madeira Islands, Portugal	CEFE Montpellier, France Acc. Number Cer3	CER	2
*maroccana*	M-*m*	*maroccana*^×^	Imouzzer, Morocco	Imouzzer, Morocco	MAR	3
*cuspidata* X *europaea*	A	*cuspidata*^×^	Grahamstown, South Africa	CEFE Montpellier Acc. Number Gr3 & Gr5	CUS	8
*cuspidata* X *europaea*	A	*cuspidata*^Ω^	Kirstenbosch, South Africa	CEFE Montpellier, France Acc. Kirstenbosch	CEH	15
Total number of genotypes						146

*All mother accessions, except “Frantoio” and “Dhokar,” are wild. ^×^Seeds obtained from different mother trees. ^Ω^Seeds obtained from the same mother tree.*

Seeds were surface-sterilized and germinated *in-vitro* in a hormone-free medium (Rugini, 1984) incubated in a growth chamber with 16 h light photoperiod (34 μM intensity with 70% red: 30% blue light-emitting diodes, LEDs, at 25 ± 2°C. Seedlings were cut into uninodal segments and micropropagated in the same Rugini medium supplemented with 1 mg/L zeatin in the same growth chamber described before. For whole plant regeneration, grown shoots were transferred to rooting medium (50% strength Rugini medium) supplemented with α-naphthalacetic acid (0.8 mg/L). Rooted seedlings were *ex-vitro* acclimatized for 3 weeks, transplanted to 2.5 L pots and then grown under greenhouse conditions.

### Morphological and Architectural Traits

Different morphological and architectural traits were evaluated on *ex-vitro* potted plants at different growing stages. In potted plants, 13 months after transplanting *ex-vitro* acclimatized seedlings, we recorded: primary shoot height; number of secondary stems; number of tertiary stems; number of total nodes; total number of leaves; basal stem diameter (measured at 5 cm above ground with a vernier caliper); and fresh weight (leaf, shoot, and root). The morphological parameters were calculated according to the following equations:

Internode⁢length=Height/Number⁢of⁢nodes⁢on⁢the⁢main⁢stem

R/A=Root⁢fresh⁢weight/Shoot⁢fresh⁢weight

Branching=Number⁢of⁢secondary⁢stems

Branchingfrequency=Numberofsecondarystems/Numberofnodes(Rosatietal.,2013)

Branchingefficiency=Numberofsecondarystems/⁢Basal⁢Diameter⁢5⁢cm⁢above⁢ground⁢(Rosatietal.,2013)

Morphological traits of 103 wild genotypes were measured in three independent experiments, using 7–10 plants per genotype (Supplementary Table S4). In order to compare the results obtained from the different assays, the GUA1 variety was grown in the three different assays to normalize the data. Ratios obtained from two parameters measured in the same plant were calculated from absolute (non-normalized) values. Correlations between vigor parameters measured in grafted plants represent the average value of 8–12 plants per grafted genotype +/− standard errors. Correlation graphics and the respective *R*^2^-values were calculated with the Excel software.

### Ploidy Level

Polyploids have been described within the *O. europaea* complex as a consequence of recent neopolyploidization events in Macaronesia (i.e., hexaploid *maroccana* and tetraploid *cerasiformis;* Besnard et al., 2008) and in the Hoggar mountains (i.e., presence of a few triploids in subsp. *laperrinei;* Besnard et al., 2008). It was thus necessary to first determine the ploidy level of each individual of the SILVOLIVE collection. This was determined by flow cytometry according to the methodology described by Aleza et al. (2009). Samples consisted of small pieces of leaves (∼ 0.5 mm^2^) collected from each genotype, which were directly compared to a well-known diploid cultivar (Córdoba WOGB, acc. number W45) as a control. Samples were chopped together using a razor blade in the presence of a nuclei isolation solution (High Resolution DNA Kit Type P, solution A; Partec ^®^, Münster, Germany). Nuclei were filtered through a 30 μm nylon filter and stained with a DAPI solution (4,6-diamine-2-phenylindol; High Resolution DNA Kit Type P, solution B; Partec ^®^). Following a 5 min incubation period, stained samples were run in a CyFlow ^®^) flow cytometer equipped with optical parameters for the detection of DAPI fluorescence at 365 nm. Histograms were analyzed using the CyView software (Partec ^®^), which determines peak position, co-efficient of variation (CV), arithmetic mean and median of the samples.

### Chloroplastic DNA Polymorphism

Genomic DNA was extracted from leaf disks using the Sigma kit REDExtract-N-AmPlant PCR. We then used plastid markers to discriminate between the different wild olive provenances in our collection [Note that three plastid lineages have been described in the Mediterranean olive (Besnard et al., 2011): lineages E1 from the eastern Mediterranean basin, and lineages E2 and E3, both from the western Mediterranean region (hereafter referred to E1, E2, and E3, respectively)]. Ten chloroplastic DNA (cpDNA) loci previously reported (Weising and Gardner, 1999; Besnard et al., 2003, 2011, 2013a; Baali-Cherif and Besnard, 2005; Besnard, 2008; Garcia-Verdugo et al., 2010) were analyzed in the present study (Supplementary Tables S1, S2).

Primers for PCR-amplification of the cpDNA markers are listed in Supplementary Table S2. Polymerase chain reactions (PCR) were performed at a final volume of 20 μL with 10 ng of template DNA, 0.5 μM primer concentration, and 2 units of MyTaq^TM^ Red DNA Polymerase (BIOLINE) through conventional PCR procedures using a BIO-RAD T100 thermal cycler. After amplification, 2 μL of the PCR product was run on a 2% agarose gel to verify amplification product size. PCR products were sequenced and chromatograms were visualized using the “Chromas” software to identify SNPs and indels. For each genotype, a final sequence was obtained through concatenation of the loci following this order: ccmp5, OeR16Qa, matK2-3, QR-1, QR-2, QR-3, trnTD-2, trnTL-1, SSR-31, and SSR-45. For each subspecies and Mediterranean lineage, we also added as a reference the same concatenated sequences extracted from full plastomes available in the NCBI database. A full chloroplastic sequence was, however, not available for subspecies *cerasiformis*. All sequences were then aligned and analyzed with the “MEGA6” software (Tamura et al., 2013). A phylogenetic analysis was performed by maximum likelihood based on the Tamura 3-parameter model (Tamura, 1992).

### Nuclear Microsatellite (SSR) Markers

Leaf samples from *in-vitro* grown seedlings were used to purify genomic DNA with the Sigma kit RED-Extract-N-AmPlant PCR. Five polymorphic nuclear SSR markers (Sefc et al., 2000) were then used to establish a genetic profile for every individual. The description of the SSR markers, including primer sequences, repetitive motif, allele size, and references are described in Supplementary Table S3. To get reference genotypes, DNA was also obtained from wild and cultivated olives maintained in different germplasm collections: subsp. *cerasiformis* (CEFE Montpellier, accession Cer3), subsp. *guanchica* (WOGB Córdoba, accession W49), subsp. *europaea* E1 (WOGB Córdoba, accession W45), subsp. *europaea* E3 (WOGB Córdoba, accession W69), as well as the cultivars “Dhokar” (WOGB Marrakech, accession Oct413) and “Frantoio” (WOGB Córdoba, accession 80). PCR reactions were performed as previously explained. SSR fragment analysis was performed with the “Peak Scanner” program (Applied Biosystems). A genotype matrix was built (Supplementary Table S3) and analyzed in R as explained below.

### Grafting

To determine grafting compatibility, the olive “Picual” and “Hojiblanca” cultivars were grafted onto 43 wild genotypes using 10 potted plants per genotype. As a control, plants from both cultivars were also grafted onto their own roots. Leaves were removed from semi-hardwood wild genotypes grown for 18 months under greenhouse conditions after *ex-vitro* acclimatization. Rootstock plantlets were cut 20 cm from the ground level. “Picual” scions with similar stem diameter, or slightly thinner than those of the rootstocks, were cut into sections containing 3–4 nodes and their leaves removed from the base. To avoid tearing the bark, two slanted downward notches were rapidly made in the basal node of the scion (characteristic tip shape). Then, a 2-cm lengthwise incision was made in the cut tip of the rootstock stem using a sharp knife to quickly insert the scion, making sure that the two sets of cambial tissue coincide. A biodegradable synthetic tape was used to seal the graft union in order to stop the entry of microorganisms and to prevent the rootstock and scion tissue cells from drying out. The grafted plant was grown for a year under greenhouse conditions before performing measurements of morphological scion features.

### R Functions and Statistical Analyses

The software R was used for different genotyping and phenotyping analyses (R: A language and environment for statistical computing. R Foundation for Statistical Computing, Vienna, Austria).^1^ For the analyses of the morphological traits, a principal component analysis (PCA) based on the correlation data matrix was performed using the package “mice” to interpolate missing values, and “factoextra” to calculate the principal components. For the analysis of nuclear markers, a genotype matrix was built considering allele sizes (Supplementary Table S3) and analyzed with the “POLYSAT” package (Clark and Jasieniuk, 2011). Then, a matrix of genetic distances was created according to Bruvo et al. (2004). The principal coordinate analysis was plotted using the “POLYSAT” package. Morphological parameters of grafted plants were represented as the average values of 8–10 plants +/– standard errors. Asterisks indicate significant differences with the self-grafted Picual plants value. The data were subjected to analysis of variance (ANOVA) and multiple comparisons of means were analyzed by Tukey’s HSD (honestly significant difference). The multiple range test was calculated using the Statistical Analysis System (STATGRAPHICS Centurion XVI software; http://www.statgraphics.com; StatPoint Technologies, Warrenton, VA, United States).

## Results

The wild olive germplasm collection SILVOLIVE includes 146 genotypes obtained from seeds of up to 120 mother trees from 19 different locations, prospected in their natural habitats or from Olive Germplasm Banks (Table 1). After *in-vitro* germination, the genotypes were micropropagated to ensure the availability of clonal plant material required for subsequent genotyping and phenotyping assays.

### Genotyping and Ploidy Level Determination of the SILVOLIVE Collection

**FIGURE 1:**
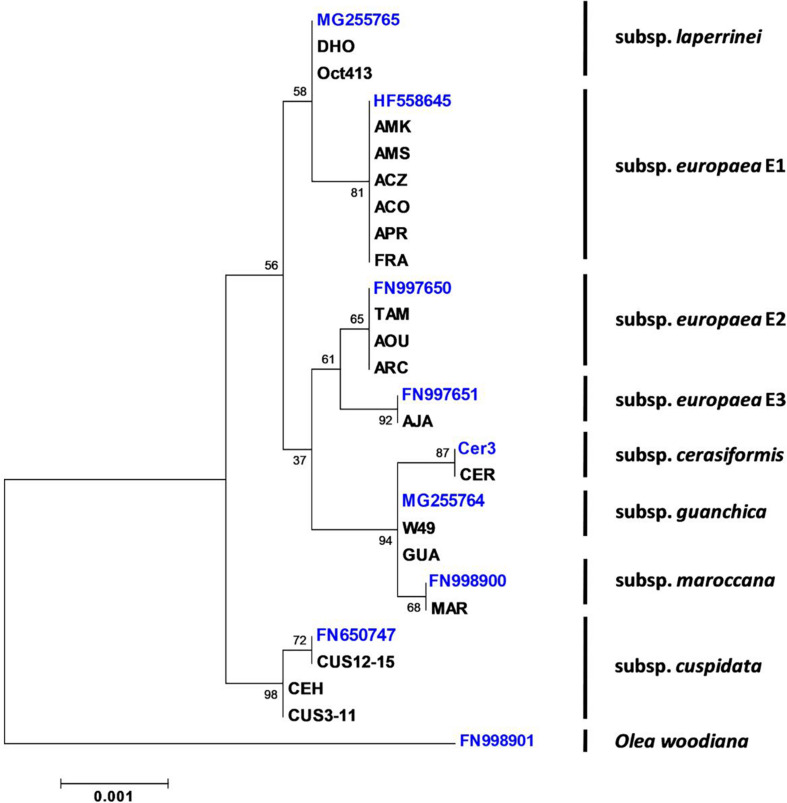
Phylogenetic relationships among wild olive genotypes of the SILVOLIVE collection according to plastid markers. Sequences of the chloroplast markers were used to determine the phylogenetic relationships through the Maximum Likelihood method based on the Tamura 3-parameter model (Tamura, 1992). Phylogenetic analyses were conducted with MEGA6 (Tamura et al., 2013). The tree with the highest log likelihood (–5551.0632) is shown. Initial tree(s) for the heuristic search were obtained by applying the Neighbor-Joining method to a matrix of pairwise distances estimated using the Maximum Composite Likelihood (MCL) approach. A discrete Gamma distribution was used to model evolutionary rate differences among sites [5 categories (+*G*, parameter = 0.05)]. The tree is drawn to scale, with branch lengths measured as the number of substitutions per site. The total number of positions in the final dataset was 3,818. Support of nodes was estimated with 1,000 bootstraps. NCBI Accession numbers of the plastomes, used here as reference genomes, are the following: *Olea europaea* subsp. *europaea* lineage E1 (HF558645); *O. e.* subsp. *europaea* lineage E2 (FN997650); *Olea europaea* subsp. *europaea* lineage E3 (FN997651); *O. e.* subsp. *laperrinei* (MG255765); *O. e.* subsp. *maroccana* (FN998900); *O. e.* subsp. *guanchica* (MG255764); *O. e.* subsp. *cuspidata* (FN650747); *O. woodiana* (FN998901). For *cerasiformis*, with no available full plastome sequence, the mother tree from the CEFE Montpellier collection (Cer3) was used to sequence the chloroplast markers. Other mother trees were also verified: W49 = *Olea europaea* subsp. *guanchica* from the WOGB Córdoba collection; Oct413 = *Olea europaea* subsp. *laperrinei* variety Dhokar from the WOGB Marrakech collection. *Olea woodiana* (FN998901) served as the outgroup species to root the tree.

To determine the origin and the degree of genetic variability of the SILVOLIVE genotypes, chloroplastic and nuclear markers have been used. Chloroplastic polymorphisms have been widely used to analyze the phylogeographic history of the olive complex (Besnard et al., 2003, 2007, 2018). According to the cpDNA markers obtained (Supplementary Tables S1, S4), the main maternal lineages of African and Mediterranean wild olives are represented in the SILVOLIVE collection (Figure 1), confirming the wide genetic variability represented in the germplasm collection. The three chloroplastic lineages previously identified in the subsp. *europaea* – as E1, E2, and E3 (Besnard et al., 2007) – were also sampled. We could also identify differences in the cpDNA of *cuspidata* genotypes. The previously fully sequenced *cuspidata* haplotype (NCBI accession number FN650747) includes the genotypes CUS12, CUS13, CUS14, and CUS15. The *cuspidata* genotypes CUS3, CUS4, CUS6, and CUS11 showed a polymorphism (C to T change) in the *trnT-trnL* spacer (Besnard et al., 2003). This attests that CUS trees (Table 1) were issued from two mothers (Gr3 and Gr5), not sharing the same chloroplastic haplotype.

**FIGURE 2:**
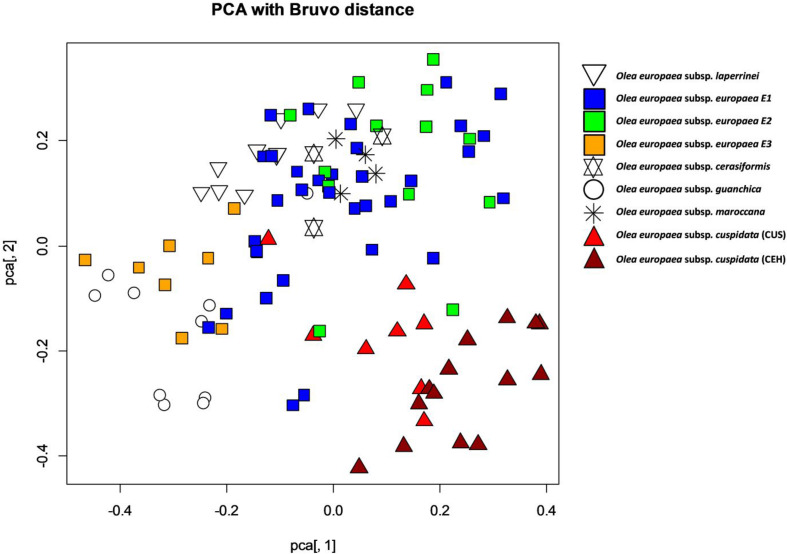
Genetic dispersion of 105 genotypes of the SILVOLIVE collection according to nuclear SSR markers. Genetic distance matrices were calculated according to Bruvo et al. (2004). To represent the distribution of the genotypes according to their genetic distances, Principal Coordinate Analysis and plotting were subsequently performed with the package “POLYSAT” in R (Clark and Jasieniuk, 2011). Plotted symbols represent different subspecies or lineages of *Olea europaea*, and different colors identified the genotypes according to the classification obtained by chloroplastic markers. To compute allele copy number, POLYSAT uses the combinatorics utilities in R (the “combn” and “permn” functions from the COMBINAT package) to match all possible combinations of alleles and find the smallest sum of geometrically transformed distances between alleles (Equation 2) of Bruvo et al. (2004).

To assess the possible genetic admixture imposed by the open pollination of the trees that gave rise to the genotypes of the SILVOLIVE collection, nuclear microsatellite markers, which are co-dominantly inherited, were analyzed. Five SSR markers were sufficient to distinguish all individuals of the SILVOLIVE collection (Supplementary Table S5). While the cpDNA markers allowed distinguishing genotypes according to their maternal origin (following to some extent the taxonomy; Besnard et al., 2018) our SSR dataset did not allow a clear distinction of taxa (Figure 2), with many SSR alleles shared between subspecies (Supplementary Table S5). Thus, individuals belonging to the same chloroplastic lineage (e.g., *europaea-*E1) exhibited a relatively large nuclear diversity (Figure 2). This may result from the admixture produced by sexual crossings of wild mother trees with pollen of genetically diverse trees present in the prospection sites, mainly in WOGBs.

Multiple polyploidy levels were revealed in the SILVOLIVE collection through flow cytometry (Supplementary Figure S1) and nuclear microsatellite analyses (Supplementary Table S5). The results showed the presence of triploids harboring the cpDNA of subsp. *europaea*-E2 (AOU10), *laperrinei* (DHO10A, DHO11A) and *cerasiformis* (CER1 and CER3); and hexaploid genotypes harboring the cpDNA of all genotypes of subsp. *maroccana*. All genotypes of *cuspidata*, *guanchica*, *europaea*-E1 and *europaea*-E3, as well as most *europaea*-E2 genotypes and *laperrinei* x *europaea* hybrids were confirmed as diploids. The presence of triploid genotypes suggests the occurrence of hybridization between diploid and polyploid genotypes in the prospecting zones, or spontaneous events of polyploidization.

### Morphological Traits of the SILVOLIVE Collection

Growth habits and vigor traits can be quantified in olive seedlings a minimum of 9 months after germination (Hammami et al., 2011). Different morphological features of root and shoot parts have thus been measured to define vigor and branching habits in 103 wild olive genotypes 13 months after *ex-vitro* acclimatization. In Supplementary Table S6, genotypes have been primarily classified according to their height because it has been described as a good trait to predict the vigor of olive plants grown in pots (De la Rosa et al., 2006). A strong phenotypic variability was observed in the height of the wild genotypes, which displayed differences of up to five times between the maximum and minimum values (Supplementary Figure S1). Among the genotypes analyzed, two hexaploids were included in the group of very reduced vigor; four triploid genotypes were distributed along the low, medium and high vigor groups; and no multiploid genotypes were present in the group of very high vigor genotypes (Supplementary Table S6). Vigor parameters, represented by the plant height showed high and positive correlations with the basal stem diameter, the shoot biomass, the total leaf biomass and the total plant biomass (Supplementary Table S7). Other group of correlated parameters comprises features characteristic of branching habits, including the branching efficiency, the branching frequency and the number of tertiary stems. The total number of nodes (and leaves) and the number of secondary stems correlated with both vigor and branching parameters. Finally, the root-to-shoot ratio showed negative correlations with both vigor and branching parameters. We observed that the high R/S ratio and the low shoot branching are traits highly represented in genotypes of very low and low vigor (Supplementary Table S8). High branching is overrepresented in the group of intermediate-vigor genotypes. Finally, the low R/S ratio is typical of genotypes with high or very high vigor (Supplementary Table S8).

**FIGURE 3:**
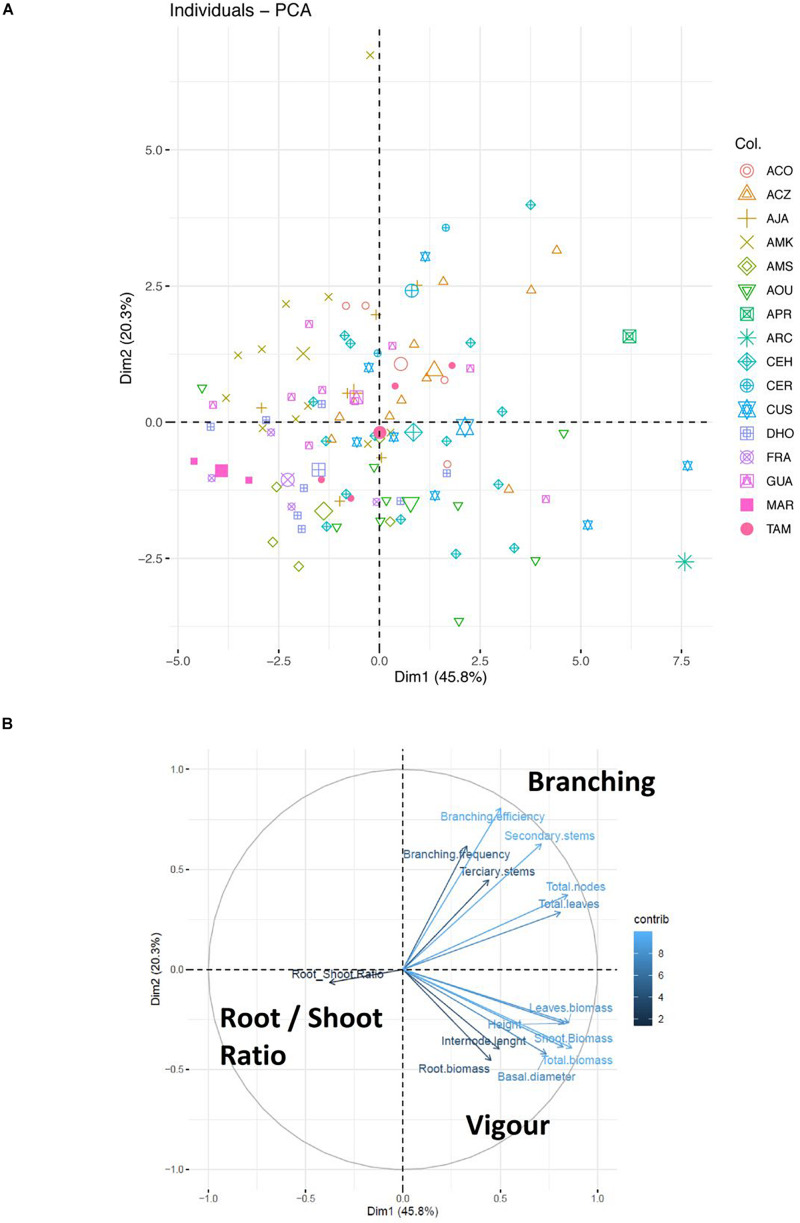
Principal Component Analysis (PCA) of morphological parameters of wild olive subspecies. Data concerning morphological parameters were converted into a matrix of numerical values. The missing data were interpolated using the “Mice” package as a preliminary step to the calculation of the main components using “Prcomp.” In **(A)** the two main components of variability, explaining here 66.5% of the total variability, were identified using the “Fviz_eig (res.pca)” algorithm and the results were plotted in a graphic. In **(B)** the contribution of each variable is depicted in the two main components previously represented in **(A)**.

According to a PCA analysis that explains 66.1% of total variability of the morphological traits measured, different groups of genotypes could be distinguished (Figure 3). ARC, AOU, CUS, and CEH genotypes are mainly characterized by high values of vigor traits: plant height, stem basal diameter, plant biomass, shoot biomass, root biomass, total leaf biomass and internode length; APR, ACZ, CER, and CEH are mainly characterized by high values of branching traits: branching efficiency branching frequency, number of secondary and tertiary stems, total number of nodes and total number of leaves; AMK, GUA, AJA, and DHO genotypes are mainly characterized by low vigor traits; MAR and DHO genotypes are mainly characterized by low branching habits and high root-to-shoot ratio. The high variability of genotypes was not only observed at the level of the whole collection, but also within the same subspecies, and even within the offspring of the same tree, as observed for example in ACZ, CUS, AOU, DHO, or CEH genotypes (Figure 3).

### Regulation of Scion Features by Wild Genotypes Used as Rootstocks

Tree grafting on clonal rootstocks is an important practice for morphological uniformity, improvement of environmental adaptability and crop quality of plants. However, it is not a widespread procedure in olive nursery production. In a first attempt to confirm the grafting compatibility of the wild genotypes with commonly used cultivars, the two high-vigor Spanish varieties “Picual” and “Hojiblanca” were grafted on 43 genotypes belonging to subspecies *guanchica, cerasiformis, laperrinei, cuspidata*, and *europaea* (lineages E1, E2, and E3) or their hybrids. All the accessions assayed, including the most genetically distant subsp. *cuspidata* showed grafting compatibility with the cultivated olive varieties used (Supplementary Table S9). Low efficient grafting compatibility was observed only for subsp. *europaea* lineage E2, for which a single genotype was tested.

**FIGURE 4:**
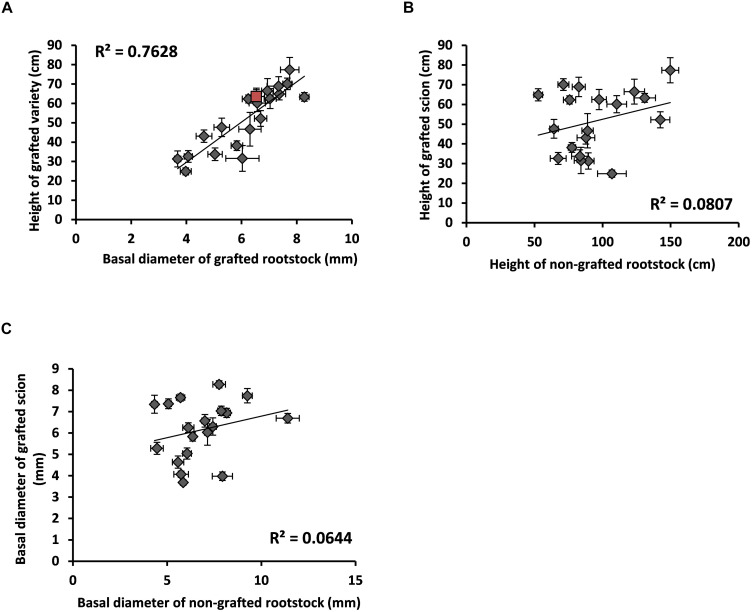
Vigor regulation of the “Picual” scion by wild rootstock genotypes. Wild genotypes grown in pots for 1 year after *ex-vitro* acclimatization were grafted with the cv. “Picual”. Morphological features were measured 1 year after grafting. **(A)** Correlation between the rootstock basal diameter and the scion height. Values correspond to the average value of different individuals (*N* = 8–12), with self-grafted “Picual” labeled in red color. **(B)** Correlation between height of the non-grafted rootstock and height of the grafted scion. Values correspond to the average value of different individuals (*N* = 8–12) of each genotype. **(C)** Correlation between the basal diameter of the non-grafted rootstock and the height of the grafted scion. Values correspond to the average value of different individuals (*N* = 8–12) of each genotype. Error bars in 2 dimensions (in **A–C**).

**FIGURE 5:**
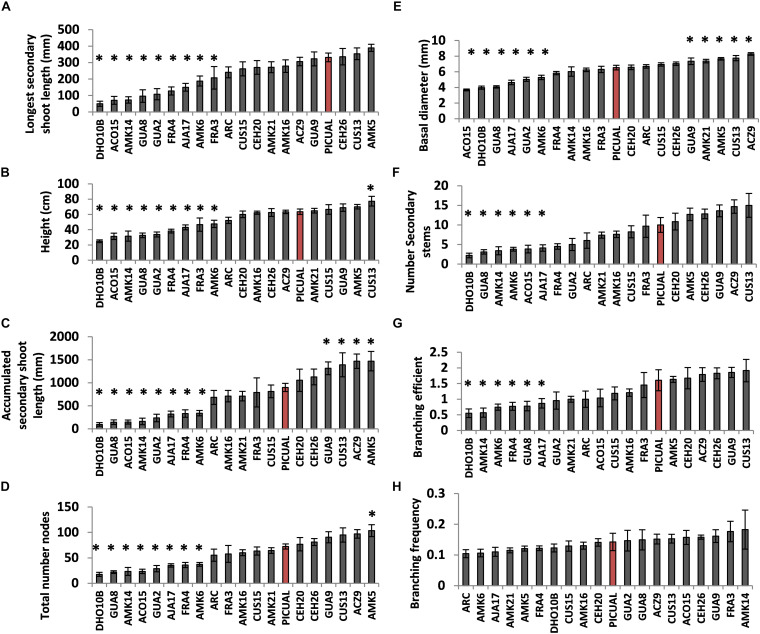
Comparison of morphological parameters measured in the Picual scion grafted on different genotypes of the SILVOLIVE collection. Potted plants grown under greenhouse conditions were used 1 year after grafting. The parameters measured were: The longest secondary shoot length **(A)**; the plant height **(B)**; the accumulated secondary shoot length calculated as the sum of all secondary shoots length **(C)**; the total number of nodes **(D)**; the basal stem diameter at 5 cm from ground **(E)**; the total number of secondary stems **(F)**; the branching efficiency **(G)**; and the branching frequency **(H)**. Bars are the average of 7–10 plants. Asterisks indicate significant differences with self-grafted Picual plants labeled as red bar. The data were subject to analysis of variance (ANOVA) and multiple comparisons of means were analyzed by Tukey’s HSD (honestly significant difference). Multiple range test was calculated using the Statistical Analysis System (STATGRAPHICS Centurion XVI software; http://www.statgraphics.com; StatPoint Technologies, Warrenton, VA, United States).

As a proof of concept, morphological traits were examined in the “Picual” scion grafted on 20 different wild olive genotypes. When used as rootstocks, many wild genotypes modified vigor parameters of the “Picual” scion (Figure 4A). As expected, most of the genotypes classified as very-low to intermediate vigor (Supplementary Table S6; DHO10B, ACO15, AMK14, GUA8, GUA2, FRA4, AJA17, AMK6, and FRA3) significantly reduced vigor properties of the grafted scion (Figures 5A–E). Conversely, high vigor genotypes such as ACZ9, CUS13 and CUS15 increased the vigor of the grafted scion (Figures 5A–E). However, some contradictory relationships were also observed. Thus, AMK5, AMK21, and GUA9, classified as very low- and low-vigor genotypes (Supplementary Table S6), significantly increased vigor parameters in the grafted “Picual” variety (Figure 5E). This indicates that not only the rootstock but also some other unknown effects (e.g., the rootstock x scion interaction) determined the scion properties in the grafted plant. As a result, when vigor traits such as basal diameter and height were compared within the same grafted plant, a positively significant correlation was observed (Figure 4A). However, when the vigor trait either height (Figure 4B) or basal diameter (Figure 4C) was compared between the grafted and the non-grafted plant, no clear correlation was observed.

While many of the tested genotypes reduced to some extent vigor parameters of the “Picual” scion (9 out of 20 genotypes showed statistically significant reductions of most vigor traits assayed; Figures 5A–F), modification of branching traits was less evident (Figures 5G,H). Most of the genotypes that reduced vigor also significantly reduced branching efficiency (Figure 5G), although no significant differences in branching frequency were observed (Figure 5F). However, FRA3, which showed significant reductions of some vigor traits, maintained similar branching efficiency, and higher (although not statistically significant) branching frequency, than the self-grafted “Picual.”

## Discussion

Ecological and socio-economic issues regarding the future of olive cultivation are essential in the light of present global changes, including agronomic, climatic, economic societal, or political changes (Besnard et al., 2018). Genetic erosion has dangerously shrunk the genetic pool of crop species. In the olive cultivation, the use of a small number of cultivars for “modern” olive orchards (e.g., in the HDH system) may lead to genetic erosion in the near future, which may increase the susceptibility of the crop to abrupt climate changes, and to the emergence of new diseases and pests (Esquinas-Alcázar, 2005). Therefore, identification of unexploited adaptive traits in wild olive genotypes and their subsequent utilization is expected to be a major goal of olive crop breeding and rootstock programmes in the close future.

### Genetic Diversity of the SILVOLIVE Collection

Germplasm characterization is a key starting point of the pre-breeding process, and molecular markers are a valuable tool for identifying and characterizing olive genotypes (do Val et al., 2012). The gain of genetic diversity in the SILVOLIVE collection was achieved at two levels: (i) firstly, at the whole collection level, it includes genotypes related to all known subspecies of *O. europaea*, that were here characterized with both plastid and nuclear markers. Besides the genetically distant subspecies *cuspidata* (Figure 1), genotypes of subspecies *europaea*, *laperrinei*, *cerasiformis*, *guanchica*, and *maroccana* belong to a monophyletic lineage from North Africa and the Mediterranean area, as previously reported (Médail et al., 2001; Rubio de Casas et al., 2006; Besnard et al., 2018); (ii) secondly, at the level of individuals, many accessions of the collection resulted from admixture between genetically distant parents. It is expected that hybrid offspring displays phenotypic performance superior to their parents due to a higher heterozygosity (Shull, 1948). The greatest manifestation of heterosis in the SILVOLIVE collection is expected to occur in genotypes resulting from the cross between *cuspidata* and *europaea* (CUS and CEH genotypes) since both parental subspecies present the maximum genetic distance (Figure 1). The admixture produced by sexual crossings of wild mother trees with pollen of diverse origin in collections or natural conditions (Figure 2), has further increased the genetic diversity and heterosis of the SILVOLIVE collection.

Different ploidy levels are also represented in the collection, first with the hexaploid *maroccana*, and second with triploids of different origins. The triploid genotypes CER1 and CER3 were obtained from a verified 4x *cerasiformis* mother tree (CEFE Montpellier, accession Cer3) and may thus result from a cross with a diploid father tree of the CEFE collection. The triploid AOU10, harboring the *europaea*-E2 chloroplast lineage, was prospected in the Southwest Morocco, where the hexaploid *maroccana* subspecies is endemic. AOU10 could be the product of a sexual crossing between a diploid female parent (*europaea*) with a hexaploid male parent (*maroccana*), although a tetraploid genotype should be rather expected. The possibility that AOU10 just resulted from a spontaneous triploidization between two oleasters cannot be ruled out (Besnard and Baali-Cherif, 2009). Similarly, the two triploid progenies issued from “Dhokar” from the WOGB Marrakech are very likely issued from a spontaneous events of polyploidization. A high level of unreduced gametes is indeed expected in “Dhokar” due to its hybrid status (Besnard et al., 2013a) that may lead to abnormalities during the formation of gametes (Mason and Pires, 2015).

### Phenotypic Diversity of the SILVOLIVE Collection

The diversity of morphological features in olive trees (shoot growth, root development, root-to-shoot biomass ratio, branching habits, total leaf surface, etc.) can be enormously relevant in terms of plant-soil interaction, plant hydraulic properties, water and nutrient uptake abilities, photosynthetic capacity, abiotic stress resistance, etc., as previously shown for a number of SILVOLIVE genotypes (Hernández-Santana et al., 2019). Illustrating the huge phenotypic variability of the collection, genotypes like DHO6A have a root biomass that approximates that of the aerial part (Supplementary Figure S2), whereas other genotypes like AMK34, AJA7 or CUS14 have a root biomass five times lower than that of their corresponding aerial biomass. Root anatomy can mediate responses to a range of abiotic and biotic stressors. In many soils, a deep-rooting, water-conserving root phenotype is likely to have several advantages (Warschefsky et al., 2016).

Besides the root development, the morphological traits measured have been organized in two groups of correlative traits, which identifies two sets of vigor and branching parameters respectively (Figure 3 and Supplementary Table S7). Particularly plant vigor traits show great variability (e.g., up to 5 times differences in height; Supplementary Figure S2). Highly vigorous phenotypes with high root biomass could be of interest to rehabilitate deforested habitats or at risk of desertification (Bekele, 2005; Kassa et al., 2019), while low-vigor genotypes could be particularly interesting for different reasons. On the one hand, they could reasonably be better suited to reduce vigor of grafted scions, which should be of great relevance for HDH olive plantations. On the other hand, 45% of very low- and low-vigor genotypes present high root-to-shoot ratio (Supplementary Table S8), which may determine a more favorable plant-soil interaction in term of stress resistance, nutrient- and water-use efficiencies. These traits may favor the reduction of irrigation and fertilizers, promoting a more sustainable agriculture. This is particularly true for very low- and low-vigor genotypes like DHO6A, AMK21, DHO12A, AMK9, GUA8, AMK12, CEH2, FRA4, DHO10B, and AMS7 (Supplementary Table S6 and Supplementary Figure S1). Most AMK genotypes (10 out of 11) are classified as low or very low vigor genotypes (Supplementary Figure S2), being a possible source of low-vigor genes. However, genotypes with low vigor are frequently discarded in olive breeding programs because of their long juvenile period (De la Rosa et al., 2006). This wide variability of vigor features was also observed in wild olive genotypes from the SILVOLIVE collection grown in the field for a longer time (León et al., 2020). Thus, similarly to the phenotype observed in potted plants, AMK genotypes showed low vigor when grown in the field, supporting the fact that morphological features observed in young plants grown in pots, like vigor, are still observed in field-grown plants. The possibility that the low-vigor AMK genotypes are inbred individuals should be bear in mind, because they could be more susceptible to adverse situations of biotic or abiotic origin.

Around 3.3 times variability was observed in the basal stem diameter of the wild olives collection. In general, genotypes with greater heights have thicker trunks, showing a positive correlation in potted plants (*R*^2^ = 0.7581), as reported also in SILVOLIVE genotypes grown in the field (León et al., 2020). However, some varieties such as FRA1, AMS15, AMK26 and DHO6A, showed thicker primary stems despite having low vigor traits according to other morphological features. The number of secondary stems also showed high variability (Supplementary Figure S1 and Supplementary Table S6), with CEH3, ACZ7, and ACZ8 exhibiting the highest number of lateral branches. The AMK26 genotype is remarkable, with the smallest size and the highest branching frequency (Supplementary Figure S1). Cultivars used for HDH orchards, such as “Arbequina” and “Arbosana,” exhibit greater branching frequency associated with smaller diameters of trunk, branches and shoots. This means low-vigor plants producing a greater number of smaller secondary stems and shoots that reduce permanent structures for a given canopy volume (Rosati et al., 2013). In this study, genotypes with low basal diameter and high branching like AMK26, CUS14, AMK27, AMK5, AJA17, AOU13, AMS12, GUA9, ACO15, ACO14, CER3, GUA4, ACZ4, and CUS3, have been identified. The branching habit is one of the main factors affecting carbon partitioning between wood and leaves. Low-vigor and high-branching genotypes of the SILVOLIVE collection have a higher number of nodes and leaves (*R*^2^ = 0.7), which means increased canopy density and may determine higher number of potential fruiting sites, becoming an important trait to be used to produce and export more assimilates toward fruits (Rosati et al., 2018). Otherwise, the hexaploid MAR1 and MAR3, together with most FRA and AMS genotypes show high apical growth with a low number of secondary stems.

### SILVOLIVE, a Germplasm Collection for the Identification of Rootstocks That Improve Olive Cultivation

Traditional olive plantations are characterized by low tree density and rain fed orchards with low yields. Progressive intensification of olive cultivation, with higher densities, irrigated, and mechanically harvested orchards has significantly increased crop productivity [e.g., higher production at lower costs; Rallo et al. (2013)]. But intensive cultivation has strongly reduced the diversity of cultivars in olive orchards, increased the demand of inputs and the risk of environment unbalances (Rallo et al., 2016) dealing, for example, to higher incidence of soil-borne diseases (Lopez-Escudero and Blanco-Lopez, 2005; Lopez-Escudero and Mercado-Blanco, 2011; Perez-Rodriguez et al., 2015). Given that grafting compatibility can occur across broad phylogenetic distances, crop wild relatives are of great significance to grafted perennial crops. Olive cultivation, specially intensive crops, could benefit from the use of clonal rootstocks, which have been proposed to potentially improve a number of agronomic traits such as: resistance to *Verticillium* wilt (Porras-Soriano et al., 2003; Bubici and Cirulli, 2012); tolerance to frost injury (Pannelli et al., 2002) and iron chlorosis (Alcantara et al., 2003); and early maturation (Malik and Bradford, 2004). But the main drawback of HDH orchards is the difficulty to control the tree size to allow the movement of the harvesting machines (Tous et al., 2010). Only a few traditional olive cultivars meet partially the low vigor requirement for HDH plantations, mostly “Arbequina,” “Arbosana,” and “Koroneiki” (Diez et al., 2016). Even these cultivars require tree size control by means of strict pruning and fertirrigation practices (Fernandez et al., 2013), which are expensive procedures. In addition, the HDH system excludes the possibility of using traditional cultivars of higher vigor, but outstanding socioeconomic importance. Rootstock-induced reduction in scion vigor, or “dwarfing” causes a decrease in tree size, reducing the need for pruning in commercial orchards. But to our knowledge, no rootstocks of proven quality are presently available at either commercial or experimental levels.

The question arises whether the phenotypic features previously described in genotypes of the SILVOLIVE collection can be somehow transmitted to the grafted scion. Plant shoot vigor is affected by numerous root-depending factors including root hydraulic pressure, water uptake efficiency, hormone production, nutrient uptake, and stomatal conductance. Convincing evidence has been provided that these traits are genetically encoded by the root portion of the grafted plant, playing the rootstock genotype essential roles in shaping variation of these traits in the scion (Warschefsky et al., 2016). In addition, rootstocks can also affect the branching pattern of the scion (Costes et al., 2010) and promote early and more abundant bearing in young olive trees (Rosati et al., 2017, 2018). On the one hand, we have verified that all subspecies can be grafted with commercial olive varieties, including *cuspidata*, which has the maximum genetic distance with *europaea* (Supplementary Table S9). On the other hand, we have also confirmed that wild genotypes used as rootstocks modify the morphological properties of the grafted scion (Figures 4A, 5). In general, a correspondence is observed between the vigor of the genotype and the vigor transmitted to the grafted scion. However, graft combinations have also been observed in which this correlation does not occur, with low-vigor genotypes increasing the vigor of the grafted scion (e.g., AMK5, AMK21, and GUA9; Figure 5E), strongly suggesting that other factors (e.g., the rootstock x scion interaction) are also relevant to determine the properties of the grafted tree. It is noteworthy that in a first trial with 20 genotypes of variable vigor, 9 genotypes significantly reduced vigor parameters of the grafted “Picual” scion (Figure 5). This represents 45% of the genotypes tested, which indicates that the ability to reduce vigor is a common trait in the collection of wild genotypes. Most of the rootstocks that reduce vigor of the “Picual” scion also significantly reduced the branching efficiency as a consequence of the reduction in the number of secondary stems (Supplementary Figure S2), given the strong correlation between both parameters (*R*^2^ = 0.93). However, no significant differences were observed in the branching frequency or average number of secondary stems per bud.

## Conclusion

This work has been carried out on plants grown in pots 1 year after grafting. It is therefore necessary to extend this study to field conditions for a greater number of years to obtain more conclusive data. For example, determining the rootstock effect on the branching frequency might require greater root development in the soil and a larger canopy volume. With this aim, field trials are currently being developed under HDH conditions with 35 selected genotypes used as rootstocks of the “Picual” and “Arbequina” scions. In addition, some of the genotypes tested have been proven resistant or extremely resistant to *Verticillium* wilt (to be published), one of the most threatening disease for olive cultivation due to the severity of damage caused and its rapid extension (Inderbitzin and Subbarao, 2014). The identification of one or several *Verticillium*-resistant dwarfing rootstocks in the collection of wild genotypes could give an important boost to the high density cultivation of olive grove, opening the possibility of: increasing the sustainability of the crop (e.g., reducing pruning and the use of fungicides); and introducing new oil or table olive cultivars (of higher vigor) into the HDH system.

Therefore, the SILVOLIVE collection, which represents an important part of the genetic variability of the species, has been genotyped and phenotyped. Great variability has been found in the wild genotypes at both genotypic and phenotypic levels. When used as rootstocks, wild genotypes modify growth parameters of the grafted scion. According to their specific characteristics, genotypes of the SILVOLIVE collection have great potential interest to: (i) restore vegetation on degraded soils or at risk of desertification; (ii) provide relevant traits or genes in breeding programs; (iii) be used as rootstocks for olive cultivation, mostly for high-density hedgerow (HDH) orchards.

## Data Availability Statement

The datasets generated for this study are available on request to the corresponding author.

## Author Contributions

PD-R participated in all experimental tasks, as well as in writing the manuscript. JF-N, JE, and CR-N participated in vigor measurement of ungrafted plants in pots. RM and AC participated in genotyping the collection with nuclear SSR markers. NC contributed in the development of the phylogenetic analyses and in the study of the genetic distances. PA contributed in determining the ploidy level of genotypes. MC and JG-F contributed in obtaining plant material and its multiplication *in vitro*. AB, LL, and GB contributed in obtaining plant material and in writing the manuscript. JC-F conceived research plans, supervised the experiments, and wrote the manuscript.

## Conflict of Interest

The authors declare that the research was conducted in the absence of any commercial or financial relationships that could be construed as a potential conflict of interest.

## Footnotes

1 https://www.R-project.org/
