# Supplementary material for: SILVOLIVE, a Germplasm Collection of Wild Subspecies With High Genetic Variability as a Source of Rootstocks and Resistance Genes for Olive Breeding
Source: Front Plant Sci. 2020 May 28;11:629. doi: 10.3389/fpls.2020.00629 (PMC7270354; doi:10.3389/fpls.2020.00629)
Supplement: Supplementary file 1 [file Data_Sheet_1.docx]

**
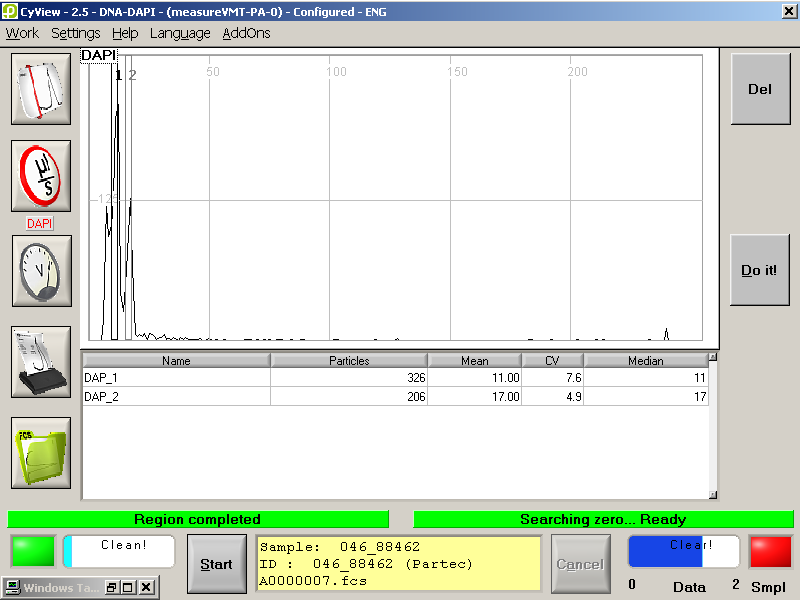

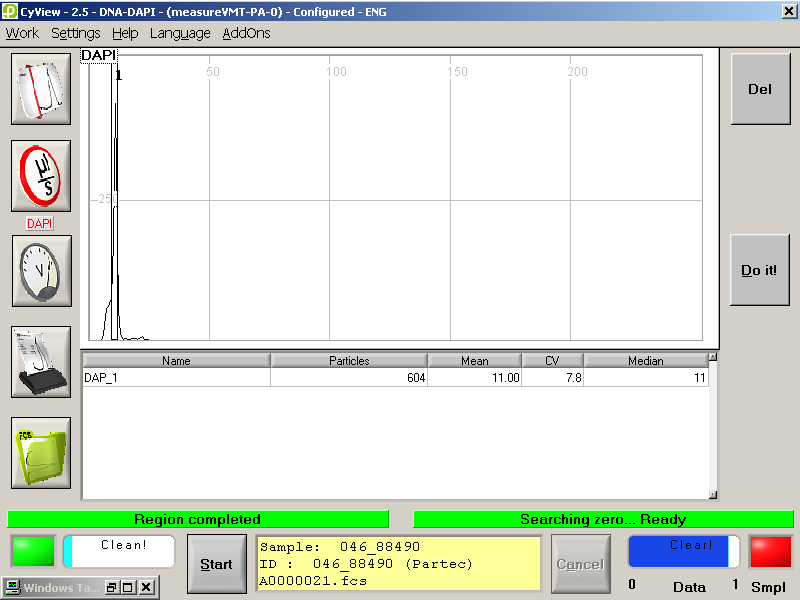

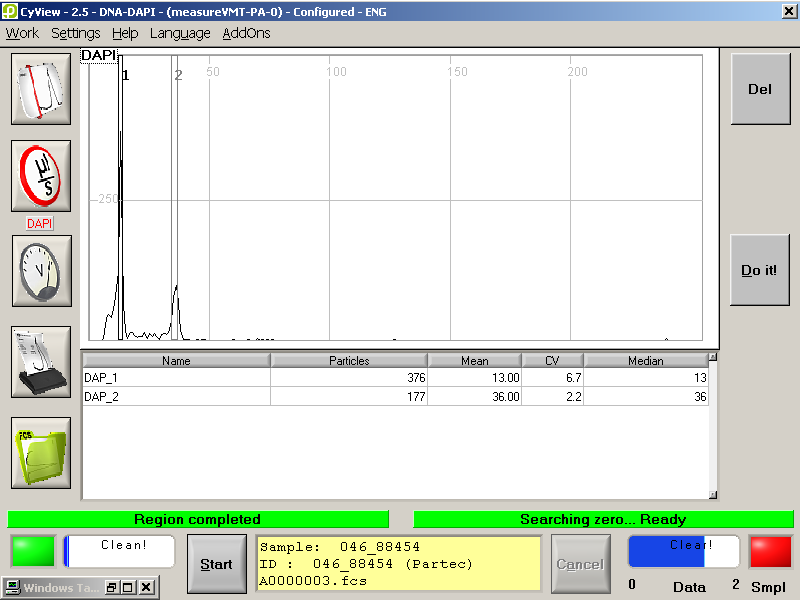
**

**Supplementary Figure S1**. Flow cytometric measurements of DAPI-stained leaves. (A) Histogram of the control diploid plant GUA1 (peak 1). (B) Histogram of the diploid control plant GUA1 (peak 1) and triploid plant AOU10 (peak 2) with index peak ratio 17/11=1.54. (C) Histogram of the diploid control plant GUA1 (peak 1) and hexaploid plant MAR2 (peak 2) with index peak ratio 36/13=2.8.

**Supplementary Figure S2**. Quantification of morphological parameters of wild olive genotypes sorted by plant height, represented in the top graph.

**Supplementary Figure S2**. Quantification of morphological parameters of wild olive genotypes sorted by plant height, represented in the top graph (continued).

**Supplementary Figure S2**. Quantification of morphological parameters of wild olive genotypes sorted by plant height, represented in the top graph (continued).

**Supplementary Figure S2**. Quantification of morphological parameters of wild olive genotypes sorted by plant height, represented in the top graph (Continued)
